# Antioxidant and Antifatigue Properties of the Aqueous Extract of *Moringa oleifera* in Rats Subjected to Forced Swimming Endurance Test

**DOI:** 10.1155/2016/3517824

**Published:** 2016-01-19

**Authors:** Bonoy Lamou, Germain Sotoing Taiwe, André Hamadou, Abene, Justin Houlray, Mahamat Mey Atour, Paul Vernyuy Tan

**Affiliations:** ^1^Department of Animal Biology and Physiology, Faculty of Science, University of Yaounde I, P. O. Box 812, Yaoundé, Cameroon; ^2^Department of Animal Biology and Physiology, Faculty of Science, University of Buea, P. O. Box 63, Yaoundé, Cameroon; ^3^Department of Biology and Sport Education, National Institute of Youth and Sports, P. O. Box 1016, Yaoundé, Cameroon

## Abstract

The effects of the aqueous extract of* Moringa oleifera* on swimming performance and related biochemical parameters were investigated in male Wistar rats (130–132 g). Four groups of rats (16 per group) were fed a standard laboratory diet and given distilled water, 100, 200, or 400 mg/kg of extract, respectively, for 28 days. On day 28, 8 rats from each group were subjected to the forced swimming test with tail load (10% of body weight). The remaining 8 rats per group were subjected to the 90-minute free swim. Maximum swimming time, glycemia, lactamia, uremia, triglyceridemia, hepatic and muscle glycogen, hematological parameters, and oxidative stress parameters (superoxide dismutase, catalase, reduced glutathione, and malondialdehyde) were measured.* Results*.* M. oleifera* extract increased maximum swimming time, blood hemoglobin, blood glucose, and hepatic and muscle glycogen reserves. The extract also increased the activity of antioxidant enzymes and decreased the blood concentrations of malondialdehyde. Furthermore, it decreased blood concentrations of lactate, triglycerides, and urea. In conclusion, the antifatigue properties of* M. oleifera* extract are demonstrated by its ability to improve body energy stores and tissue antioxidant capacity and to reduce the tissue build-up of lactic acid.

## 1. Introduction

Fatigue is best defined as difficulty in initiating or sustaining voluntary activities [1]. Fatigue is accompanied by a feeling of extreme physical or mental tiredness, resulting from severe stress and hard physical or mental work [2]. Physical fatigue is thought to be accompanied by deterioration in performance [1, 3]. There are several theories about the mechanisms of physical fatigue. These include the exhaustion theory, the clogging theory, the radical theory, and the hemoglobin theory. The exhaustion theory suggests that during exercise, many energy sources, such as glucose and liver glycogen, will be exhausted, thus leading to physical fatigue [4]. Prolonged, continuous utilization of carbohydrates depletes hepatic and muscle glycogen reserves (at 60 to 80% of maximal oxygen consumption (VO_2_max)) and considerably diminishes blood glucose concentration to subphysiological concentrations, resulting in fatigue [5, 6]. The radical theory suggests that intense exercise can produce an imbalance between the body's oxidation system and its antioxidation system. The accumulation of reactive-free radicals will put the body in a state of oxidative stress and bring injury to the body by attacking large molecules and cell organs. The mechanisms and cellular systems responsible for oxidative stress include mitochondria, leucocytes, and ischemia-reperfusion, and recovery from exercise-induced fatigue requires damage repair and elimination of the accumulated metabolic products [1, 4, 7–10]. The clogging theory suggests that exercise can cause the production and accumulation of metabolism-related substances such as lactic acid and urea in the body, which affect cellular homeostasis. The resulting acute or chronic acidosis triggers muscle cramps, muscular pain, acute respiratory distress, inhibition of enzymatic systems, and eventually fatigue [11–13]. The hemoglobin theory on its part suggests that myoglobin and an energy metabolic system coenzyme leak out into the blood from cells and tissues damaged by exercise, and destruction of red blood cells occurs [14].

*Moringa oleifera* Lam. is a plant of Indian origin which is now widespread in Asia and Africa. It belongs to the family Moringaceae with about 13 species [15]. It is commonly referred to as “tree of life,” “miracle tree,” or “divine plant” due to its numerous nutritive, medicinal, and industrial potentials [16, 17]. The leaves are widely consumed as a legume and used in traditional medicine in Africa in general and in Cameroon in particular. The leaves are an excellent source of protein (19–35% dry matter) [18–26] and are rich in metabolisable energy (2273–2978 kcal/kg DM) [18, 25], vitamins (A, B, C, and E), minerals (0.6–11.2% DM), for example, iron, calcium, zinc, selenium, and *β*-carotene [19, 27], and fats (2.3–10% DM) and contain the 10 amino acids essential to man [19, 20, 24, 25]. Crude protein levels of 30.3% and 19 amino acids have been reported in the South African ecotype of the plant [28].* M. oleifera* leaves are used in traditional medicine to treat malnutrition, fever, headaches, nerve pain, and diabetes [24, 29]. The leaves are used in Benin as food supplements for HIV patients [30] and as antipyretic and antibiotic [31]. In part one of a comprehensive review of the medical evidence for its nutritional, therapeutic, and prophylactic properties,* M. oleifera* has been cited for its numerous applications in disease treatment and prevention. These include antibiotic, antitrypanosomal, hypotensive, antispasmodic, antiulcer, anti-inflammatory, hypocholesterolemic, and hypoglycemic activities, as well as having considerable efficacy in water purification by flocculation, sedimentation, antibiosis, and even reduction of schistosoma cercariae titer. The plant family is rich in compounds containing the simple sugar, rhamnose, and also rich in a fairly unique group of compounds called glucosinolates and isothiocyanates, some with anticancer activity [32, 33]. Torres-Castillo et al. [34] have provided an overview of the histological organization and the composition of some biochemical components (e.g., enzymatic inhibitors, phytochemicals, enzymatic profiles, and antifungal potential) of different plant tissues of* M. oleifera*, associating these components with the physiology and defense mechanisms of the plant.

Nutrient supplementation to improve exercise performance has involved the use of high fat diets, carbohydrate supplements, and various dietary supplements or “tonics” of plant origin to enhance exercise capacity. In the fight against fatigue, more and more sports professionals and athletes are turning to plant extracts as sources of energy in replacement of banned doping substances. Studies have revealed the widespread use of plant extracts in many African countries for performance enhancement [35]. It is therefore important to develop efficient and safe plant-based antifatigue products that can enhance exercise performance without deleterious effects on the health of the users. It has been widely claimed that “ounce-for-ounce, Moringa leaves contain more Vitamin A than carrots, more calcium than milk, more iron than spinach, more Vitamin C than oranges, and more potassium than bananas” and that the protein quality of Moringa leaves rivals that of milk and eggs. Given the rich nutrient, phytochemical, and organoleptic potential of* M. oleifera*, we designed the present experiment to study the antifatigue potency of the aqueous extract in rats subjected to the forced swimming test.

## 2. Materials and Methods

### 2.1. Materials

#### 2.1.1. Plant Material and Preparation of Aqueous Extract of Leaves from* M. oleifera*

The fresh leaves of* M. oleifera* were harvested from the North Region of Cameroon in December 2014 and identified in the National Herbarium (Yaoundé) where a voucher specimen No. 49178/HNC exists. The leaves were cleaned immediately after harvest, cut into small pieces, and dried in the shade for about 2 weeks. The dried material was ground into a powder using an electrical homogenizer (Zaiba*®*). The aqueous extract was prepared as described by Thilza et al. [36]. 100 g of ground plant material was macerated in 1.5 L of boiled distilled water for one hour. The mixture was filtered through Whatman filter paper No. 3 and filtrate obtained was evaporated to dryness using a rotator evaporator at 45°C. The extract obtained (22, 9% yield) was stored at 4°C. Extract solution was prepared in distilled water each time prior to experimentation.

#### 2.1.2. Animals and Grouping

*(1) Animals*. Male albino rats of Wistar strain weighing 130–132 g were obtained from the Animal House of the National Institute of Youth and Sports in Yaoundé. They were placed in plastic cages in a room under standard laboratory conditions (temperature 20 to 30°C, relative air humidity 45 to 55%, and 12/12 h light/dark cycle). The rats were fed with a basal diet and water* ad libitum*. The feed was a standard rat chow (National Veterinary Laboratory (LANAVET), Cameroon) composed of carbohydrates (52%), protein (22%), fat (6.5%), water (12%), ash (6%), and fiber (4.5%). The authorization for the use of laboratory animals in this study was obtained from the Cameroon National Ethics Committee (Reg. No. FWA-IRBoooo1954). The use, handling, and care of animals were done in adherence to the European Convention (Strasbourg, 18.III.1986) for the protection of vertebrate animals used for experimental and other purposes (ETS-123), with particular attention to Part III, articles 7, 8, and 9. The animals were transferred to the laboratory at least 1 hour before the start of the experiment. The experiments were performed during the day (11:00–17:00 hr).

*(2) Animal Grouping.* After two weeks of acclimatization, sixty-four rats were divided randomly into four groups of sixteen rats each: group 1: a control group which received the vehicle (distilled water) only, and three treatment groups (groups 2, 3, and 4) which received 100, 200, and 400 mg/kg, respectively, of* M. oleifera* extract orally once a day for 28 days. The quantity of food and water consumed by each group of rats, as well as body weights were measured every two days during 28 days, between 11:00 AM and 12:00 AM before extract administration.

### 2.2. Methods

#### 2.2.1. Weight Loaded Force Swimming Test

The weight loaded force swimming test was performed as described previously [37–41] but with some modifications. Briefly, 30 minutes after the last dose of extract on day 28 of treatment, eight rats taken from each group were subjected to the force swimming exercise. Each animal was supplied with a constant load (corresponding to 10% of the body weight) tagged to the tail and placed individually in a swimming pool (90 cm × 45 cm × 45 cm), filled with water to a depth of 35 cm [42, 43] and maintained at 25 ± 1°C [41]. Exhaustion was determined by observing loss of coordinated movements and failure to return to the surface within 10 sec [41, 44] and swimming time was recorded immediately. The rats were then removed from the pool, dried with a paper towel, and returned to their original cages. The pool water was replaced after each session.

#### 2.2.2. The 90-Minute Free Swimming Test

Thirty minutes after the final extract treatment, the remaining eight rats from each group were subjected to the 90-minute free swimming experience without a weight load. At the end of the swim, the rats were rested for an hour and then sacrificed (under ether anesthesia) by cutting through the jugular vein. Blood samples were taken into sterile tubes and serum was prepared (centrifuging at 3000 rpm for 10 min) for the analysis of blood glucose (Glu), triglycerides concentration (TG), blood lactic acid (BLA), and blood urea nitrogen (BUN). Another part of blood collected into bottles containing EDTA as anticoagulant was used to determine blood cell count. Vital body organs (spleen, fatty mass, heart, lungs, kidneys, and testicles) were cleaned using 0.9% saline and then weighed using a sensitive electronic balance. Tissue samples of liver and gastrocnemius muscle were taken and stored frozen at −20°C awaiting determination of glycogen and antioxidant status parameters.

#### 2.2.3. Measurement of Serum Biochemical Parameters and Blood Cell Count

Blood cell count in all rat groups was measured with the help of blood analyzer (Hospitex Diagnostic Hema Screen 18). The serum levels of glucose were estimated using a glucometer (Reader Accu-CHEK*®* Active). Blood lactic acid concentrations and levels of BUN were measured using an L-lactate assay kit (Abcam 65331 L-Lactate assay kit) and a colorimetric and enzymatic method (Bioassay System, CA Kit), respectively. Triglyceride concentrations were measured using a commercial kit for measurement of triglycerides in serum or plasma (Enzymatic Trinder Method).

#### 2.2.4. Measurement of Tissue Glycogen

Liver and muscle glycogen contents were measured calorimetrically using anthrone reagent [45]. Briefly, after hydrolysis of the liver and gastrocnemius muscle samples in 30% KOH at 100°C for 30 min, 1.5 mL of anhydrous ethanol was added to the vials. After centrifugation at 4000 ×g for 15 min, the supernatants were discarded. 0.5 mL of distilled water and 1 mL of 0.2% anthrone were added, and the vials were placed in a boiling water bath for 20 min. The absorbance of the solution in vials was determined at 620 nm using a spectrophotometer (V-530, Jasco Co., Japan).

#### 2.2.5. Measurement of Antioxidant Status in Liver and Gastrocnemius Muscle

The liver and gastrocnemius muscle tissue (1 g of each) was homogenized in 4 mL of Tris/HCl. These tissue homogenates were centrifuged at 4000 g for 15 min at 4°C and the supernatants were assessed for the antioxidant status. Lipid peroxidation (the level of thiobarbituric acid reactive substances in terms of malondialdehyde) was measured as described previously [46], and total glutathione (GSH) content was measured according to the method of Ellman [47]. Catalase activity was measured according to the method of Sinha [48]. Superoxide dismutase (SOD) activity was measured using the pyrogallol autoxidation method [49].

#### 2.2.6. Statistical Analysis

Statistical analysis was done by one-way analysis of variance (ANOVA) followed by Dunnett's test for multiple comparisons and *P* values less than 0.05 were considered significant. The results are expressed as mean ± standard error of mean (SEM).

## 3. Results and Discussion

**Figure 1:**
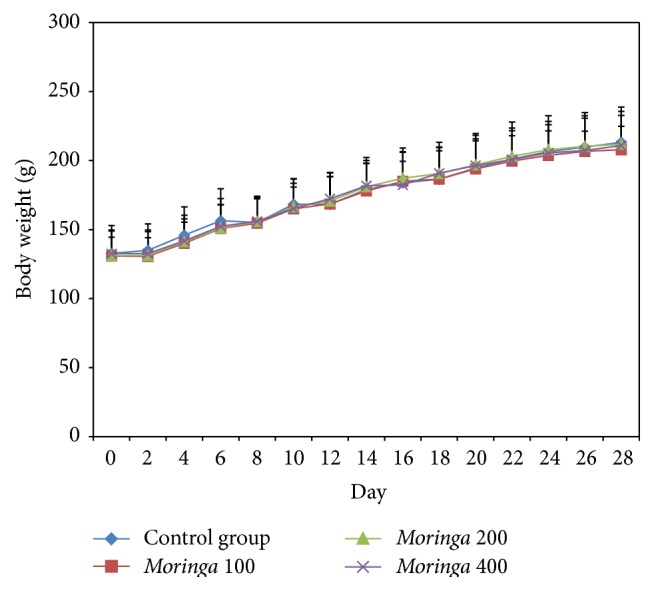
Body weight change of the rats during the experimentation. The values are expressed as mean ± SEM, *n* = 16. Control group = rats treated with distilled water;* Moringa* 100,* Moringa* 200, and* Moringa* 400 group = rats treated with 100, 200, and 400 mg/kg, respectively, of* Moringa oleifera* extract.

The forced swimming test represents a valid animal model for screening antifatigue potency of various bioactive compounds [3, 50–52]. Administration of* M. oleifera* extract did not bring about significant differences in food and water intake (Table 1). In addition, we did not observe significant differences in final body weights (207–213 g) (Figure 1) and organ weights (Table 2) following 28 days of extract administration. Body weight gain ranged between 77.0 and 80.8 g for the four treatment groups. This result was in contrast to the results obtained by Osman et al. [53] who reported up to 14% changes in body weight of rats given* M. oleifera* extract for 21 days, attributing these changes to the rich nutrient quality of the extract.

**Figure 2:**
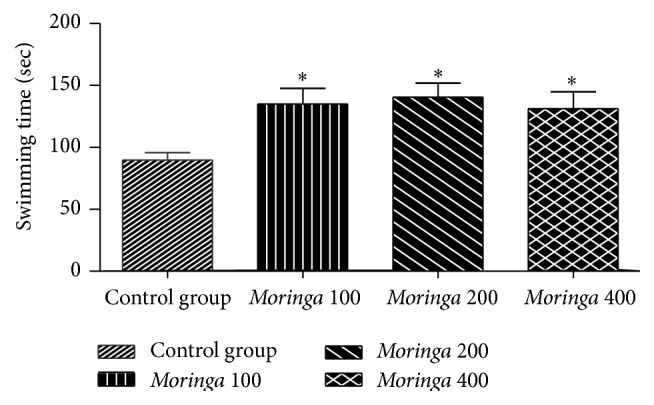
Effect of the* M. oleifera* aqueous extract on swimming time in rats. Data are presented as mean ± SEM, *n* = 8. Control group = rats treated with distilled water;* Moringa* 100,* Moringa* 200, and* Moringa* 400 group = rats treated with 100, 200 and 400 mg/kg of* M. oleifera* extract, respectively. ^*∗*^ *P* < 0.05 compared with control group.

In this study, the forced swimming capacity test in mice was employed to evaluate the effect of leaf aqueous extract from* M. oleifera* on exercise durability of rats with 10% tail load. The forced swimming capacities are shown in Figure 2. The results showed that the swimming time to exhaustion of each extract-treated group was significantly longer (*P* < 0.05) than that recorded for the control group. The maximum forced swimming times were 135.12 ± 35.62, 140.5 ± 32.17, and 131.25 ± 38.64 seconds, respectively, for the 100, 200, and 400 mg/kg extract-treated groups compared with 89.75 ± 17.19 seconds for the control group. The 200 mg/kg dose was most effective. The shortness of the length of the exhaustive swimming time indicates the degree of fatigue [54]. The results therefore indicated that extract of* M. oleifera* enhanced the swimming capacity by delaying the onset of physical fatigue in rats. Similar results have been obtained by other workers who tested the antifatigue potential of various plant extracts [3, 50–52].

Serum biochemical parameters are shown in Table 3. Results show that after the swimming test, blood glucose levels were significantly and dose-dependently higher (*P* < 0.05–*P* < 0.01) in the extract-treated groups compared with the controls. On the contrary, blood lactate levels were significantly (*P* < 0.05–*P* < 0.01) and dose-dependently reduced (20.9–36.7%) by extract treatment compared with the controls. Blood lactate is the glycolysis product of carbohydrates under anaerobic conditions and glycolysis is the main energy source for intense exercise over a short time. Therefore, lactate concentrations serve as indicators for judging the intensity of the exercise or the degree of fatigue. With the accumulation of blood lactate, blood and muscle tissue pH reduces, a condition which is harmful to some vital organs and which also causes fatigue [55–57]. Blood lactate levels are therefore representative of the degree of postexercise fatigue and the condition of recovery [44, 58]. Antifatigue agents have been shown to effectively work by delaying lactate accumulation either by reducing the glycolytic process or by increasing the rate of removal of blood lactate [56, 59]. The leaf extract of* M. oleifera* could effectively delay the onset of fatigue through one or both of these mechanisms.

The importance of muscle glycogen levels in endurance exercise has been demonstrated and it is suggested that depletion of muscle glycogen is an important factor in fatigue and exhaustion [60]. However, there is evidence that energy provision for intense prolonged aerobic muscular work relies mainly on fat utilization [61]. In this study, plasma TG levels were significantly lowered (*P* < 0.05) in all extract-treated groups compared with the controls, while extract treatment raised blood glucose levels (Table 3). In addition, following the swim test, extract-treated rats at all dose levels maintained significantly higher concentrations of muscle and hepatic glycogen compared with the controls (Table 4). Hepatic and muscle glycogen reserves constitute reliable determinants of fatigue on which endurance capacity relies [62], and the prolonged exercise-induced hypoglycemia can be harmful to nervous function [63, 64]. The concomitant significant drop in serum triglyceride concentrations and elevated blood glucose levels after endurance exercise suggest that* M. oleifera* extract preferentially promoted the utilization of fat during prolonged exercise, a glycogen-sparing mechanism that delays the onset of fatigue [65, 66]. There is experimental evidence that endurance can be improved by increasing the availability of fatty acids and that this effect is mediated by a slowing of glycogen depletion [67].

Blood urea nitrogen (BUN) levels are shown in Table 3. BUN concentrations of extract-treated rats (26.4–28.2 mg/dL) were significantly lower (*P* < 0.01) following the experimental swimming exercise compared with controls (45.0 mg/dL). The positive correlation between BUN levels and the degree of exercise tolerance is well known [60, 68, 69]. The less adapted or tolerant the body is to prolonged exercise, the more significant the rise in BUN levels following protracted exercise [70, 71]. The results therefore suggest that treatment with* M. oleifera* extract for 28 days can contribute to fatigue retardation by reducing hepatic amino acid and protein catabolism during exercise.

Intense physical exercise also causes oxidative stress in the body due to excessive generation of oxygen-derived free radicals. During exercise, a large amount of oxygen is consumed and 4-5% of the total oxygen consumed during respiration is incompletely reduced to water and therefore results in the acceleration of free radical generation. These radicals, in turn, oxidatively degrade biomolecules such as lipids, proteins, and nucleic acids and therefore affect the homeostatic environment of cells. A vast amount of evidence indicates that reactive oxygen species (ROS) are responsible for exercise-induced protein oxidation and contribute strongly to muscle fatigue [72]. As shown in Table 5, MDA concentrations in liver and gastrocnemius muscle of rats treated with* Moringa* extract were significantly lower (*P* < 0.05) compared with the controls, while the activities of SOD, GPx, and CAT in liver and gastrocnemius muscle of control rats were significantly lower (*P* < 0.05) compared with the extract-treated groups. MDA is one of the degradation products in the lipid peroxidation process [71]. Earlier studies have shown that lipid peroxidation in liver and muscle tissues increases during intense physical exercise [44]. Peroxidation is an important indicator of oxidative stress that results from degradation of cell membrane by free radicals. The results of the present study indicated the antiperoxidation capacity of* M. oleifera* extract. Enzymatic antioxidant systems, such as GPx, SOD, and CAT, are important in scavenging free radicals and their metabolites [73]. SOD protects cells by catalyzing the conversion of superoxide radicals to O_2_ and H_2_O_2_. This toxic H_2_O_2_ is further decomposed into O_2_ and H_2_O by catalase. GPx catalyzes the reduction of hydroperoxides by glutathione. These antioxidant defense mechanisms become weaker during chronic fatigue and other disease conditions [1, 72]. Thus, the improvement in the activities of these defense mechanisms can help to fight against fatigue. Our results indicated that the antifatigue effect of* M. oleifera* extract probably occurs through protection of corpuscular membranes by preventing lipid oxidation via modifying activities of several enzymes. These results are in accordance with the findings by Wang and Yan [74], which demonstrated similar effects of ginseng polysaccharides on MDA and GPx levels. Extract effect was maximal at doses 100, 200, and 400 mg/kg, respectively, for blood hemoglobin (Table 6), hepatic/muscle glycogen (Table 4), and blood glucose concentrations (Table 3). However, the dose-dependent increase in blood glucose levels was not translated into a corresponding dose-dependent increase in maximum swimming time which peaked at dose 200 mg/kg. This limited response may be explained by the effect of extract on hepatic and muscle antioxidant parameters which peaked at dose 200 mg/kg (Table 5). These results respond to the notion that antioxidants can paradoxically become prooxidant when administered at excessive doses. It is for this reason that food-derived antioxidants are preferably taken in the form of a composite mixture of many antioxidants with complimentary activity rather than a massive supply of a single antioxidant [75]. The results obtained here may therefore reflect an excessively massive import of a high concentration of one or more potent antioxidant components of the extract at the dose of 400 mg/kg.

Hematological parameters of the rats measured after exercise are presented in Table 6. There were significant increases (*P* < 0.05) in hemoglobin (Hb) and percentage lymphocytes in rats given 100 mg/kg of extract compared with the controls. Hemoglobin is the main component of erythrocytes whose main function is to serve as the carrier for oxygen and carbon dioxide. Hb also plays a role in the maintenance of the body fluid's acid/alkali balance [76]. Therefore, it can directly affect energy metabolism, body function, and exercise ability, the loading capacity of the exercise and consequently fatigue [77]. Hb normally is one of the indicators that reflect the degree of recovery from fatigue after exercise, and higher levels of Hb can improve exercise ability [44]. Our results are in accordance with the findings by Okwari et al. [78], who demonstrated similar effects of the leaf aqueous extract of* M. oleifera* on hemoglobin levels in rats subjected to thermooxidized palm oil diet-induced toxicity. Table 6 also shows that extract treatment also decreased hematocrit, percentage of granulocyte, and lymphocytes at 100 and 200 mg/kg doses (*P* < 0.05). Many studies describe changes induced by physical exercise on subtypes of blood mononuclear cells (neutrophiles, lymphocytes, and monocytes) [79, 80]. In general, during and immediately after intense exercise, total circulating numbers of leucocytes (polynuclear and mononuclear) increase in proportion to the intensity and the duration of the exercise [81] but disappear 24 hours after the exercise.

In conclusion, the leaf aqueous extract of* M. oleifera* possesses antifatigue properties. It improved the swimming ability of rats by delaying the accumulation of blood lactate and blood urea nitrogen, by increasing the mobilization and use of body fats, and by slowing the depletion of glycogen stores. The antifatigue potential may be expressed through mechanisms that involve the antioxidant activity of the extract. Further studies are needed to determine the effect of the extract on chronic physical activity.

## Figures and Tables

**Table 1:** Effect of *M. oleifera* extract on food (g/group/week) and water (mL/group/week) intake in rats.

	Control	*M. oleifera* (100 mg/kg)	*M. oleifera* (200 mg/kg)	*M. oleifera* (400 mg/kg)
Food intake				
Week 1	327.67 ± 40.81	252.00 ± 37.40	269.00 ± 32.08	319.33 ± 19.03
Week 2	285.67 ± 14.64	231.33 ± 4.93	257.67 ± 17.62	304.00 ± 42.33
Week 3	299.67 ± 16.86	243.67 ± 33.86	232.33 ± 35.53	290.00 ± 28.58
Week 4	335.00 ± 22.01	249.25 ± 29.65	286.25 ± 29.77	321.75 ± 37.85
Water intake			
Week 1	341.33 ± 33.50	319.00 ± 14.8	341.00 ± 30.51	322.67 ± 24.42
Week 2	396.67 ± 16.77	343.00 ± 34.65	381.33 ± 14.74	390.00 ± 38.43
Week 3	332.33 ± 46.01	285.33 ± 19.55	337.67 ± 26.63	308.67 ± 12.22
Week 4	346.25 ± 26.36	322.01 ± 31.76	353.02 ± 16.58	323.75 ± 33.63

Each value represents the mean ± SEM, *n* = 8.

**Table 2:** Effect of *M. oleifera* extract on relative organ weights of the rats.

Organ weight (% body weight)	Control	*M. oleifera *(100 mg/kg)	*M. oleifera *(200 mg/kg)	*M. oleifera *(400 mg/kg)
Heart	0.28 ± 0.04	0.30 ± 0.04	0.29 ± 0.05	0.34 ± 0.04^*∗*^
Liver	3.74 ± 0.27	3.24 ± 0.69^*∗*^	3.38 ± 0.24	3.53 ± 0.17
Lungs	0.73 ± 0.12	0.93 ± 0.23	0.89 ± 0.41	0.85 ± 0.07
Spleen	0.24 ± 0.06	0.32 ± 0.08	0.28 ± 0.17	0.34 ± 0.09
Left kidney	0.28 ± 0.03	0.27 ± 0.05	0.28 ± 0.03	0.31 ± 0.04
Right kidney	0.30 ± 0.02	0.26 ± 0.04	0.26 ± 0.05	0.32 ± 0.06
Left testis	0.50 ± 0.14	0.47 ± 0.08	0.61 ± 0.14	0.59 ± 0.08
Right testis	0.55 ± 0.14	0.44 ± 0.10	0.51 ± 0.07	0.59 ± 0.07
Fatty mass	1.65 ± 0.32	1.43 ± 0.55	2.27 ± 1.07	1.67 ± 0.37

Each value represents the mean ± SEM, *n* = 8. ^*∗*^ *P* < 0.05 compared with control group.

**Table 3:** Effect of *M. oleifera* extract on rat serum biochemical parameters.

	Control	*M. oleifera* (100 mg/kg)	*M. oleifera* (200 mg/kg)	*M. oleifera* (400 mg/kg)
Glycemia (mg/dL)	119.9 ± 24.87	145.01 ± 16.25^*∗*^	147.4 ± 21.35^*∗*^	163.9 ± 16.10^*∗ ***∗∗**^
Lactamia (mmol/L)	31.27 ± 3.21	24.14 ± 2.41^*∗*^	19.37 ± 4.53^*∗ ***∗∗**^	18.87 ± 6.77^*∗ ***∗∗**^
Urea (mg/dL)	45.03 ± 13.26	26.78 ± 8.77^**∗∗**^	26.41 ± 8.19^**∗∗**^	28.21 ± 9.37^**∗∗**^
Triglycerides (mg/dL)	176.77 ± 26.61	75.05 ± 7.63^*∗ ***∗∗**^	125.28 ± 12.40^*∗ ***∗∗**^	58.42 ± 7.22^*∗ ***∗∗**^

Each value represents the mean ± SEM, *n* = 8. ^*∗*^ *P* < 0.05, ^**∗∗**^ *P* < 0.01, and ^*∗ ***∗∗**^ *P* < 0.001 compared with control group.

**Table 4:** Effect of *M. oleifera* extract on rat hepatic and muscle glycogen.

Groups	Glycogen (mg/g)	
	Liver	Muscle
Control	17.68 ± 1.74	1.19 ± 0.46
*M. oleifera* (100 mg/kg)	21.57 ± 2.45^*∗*^	2.06 ± 0.77^*∗*^
*M. oleifera* (200 mg/kg)	22.69 ± 3.44^**∗∗**^	2.76 ± 0.78^*∗ ***∗∗**^
*M. oleifera* (400 mg/kg)	22.64 ± 3.69^**∗∗**^	2.68 ± 0.68^*∗ ***∗∗**^

Each value represents the mean ± SEM, *n* = 8. ^*∗*^ *P* < 0.05, ^**∗∗**^ *P* < 0.01, and ^*∗ ***∗∗**^ *P* < 0.001 compared with control group.

**Table 5:** Effect of *M. oleifera* extract on rat hepatic and muscle antioxidant parameters.

	Control	*M. oleifera* (100 mg/kg)	*M. oleifera* (200 mg/kg)	*M. oleifera* (400 mg/kg)
Hepatic parameters				
SOD (U/mg protein)	31.09 ± 3.15	35.35 ± 2.61^*∗*^	38.42 ± 3.22^*∗ ***∗∗**^	39.11 ± 3.48^*∗ ***∗∗**^
MDA (*μ*mol/g)	52.00 ± 5.66	42.13 ± 3.64^*∗ ***∗∗**^	41.63 ± 4.91^*∗ ***∗∗**^	42.32 ± 4.43^*∗ ***∗∗**^
CAT (U/mg protein)	25.13 ± 2.17	27.52 ± 1.64	29.33 ± 1.92^**∗∗**^	28.34 ± 2.67^*∗*^
GPx (*μ*mol/mg protein)	3.62 ± 0.71	5.63 ± 1.13^**∗∗**^	6.22 ± 1.12^*∗ ***∗∗**^	6.95 ± 1.74^*∗ ***∗∗**^
Muscle parameters				
SOD (U/mg protein)	6.55 ± 0.98	8.95 ± 1.06^*∗ ***∗∗**^	9.23 ± 1.54^*∗ ***∗∗**^	9.12 ± 0.87^*∗ ***∗∗**^
MDA (*μ*mol/g)	33.24 ± 5.24	24.13 ± 4.57^**∗∗**^	22.28 ± 4.63^*∗ ***∗∗**^	22.14 ± 4.54^*∗ ***∗∗**^
CAT (U/mg protein)	0.27 ± 0.05	0.32 ± 0.04	0.38 ± 0.03^*∗ ***∗∗**^	0.39 ± 0.05^*∗ ***∗∗**^
GLU (*μ*mol/mg de protein)	0.88 ± 0.37	2.98 ± 0.26^**∗∗**^	4.09 ± 1.97^*∗ ***∗∗**^	3.75 ± 1.04^*∗ ***∗∗**^

Each value represents the mean ± SEM, *n* = 8. ^*∗*^ *P* < 0.05, ^**∗∗**^ *P* < 0.01, and ^*∗ ***∗∗**^ *P* < 0.001 compared with control group.

**Table 6:** Effect of *M. oleifera* aqueous extract on hematological parameters of rats.

	Control	*M. oleifera* (100 mg/kg)	*M. oleifera* (200 mg/kg)	*M. oleifera* (400 mg/kg)
WBC ×10^3^/mm^3^	10.70 ± 4.45	9.08 ± 1.67	9.60 ± 2.47	11.32 ± 4.31
RBC ×10^6^/mm^3^	6.52 ± 0.25	6.57 ± 0.70	6.13 ± 0.16	6.3575 ± 0.2
Hb (g/dL)	12.30 ± 1.01	13.00 ± 0.22^*∗*^	12.38 ± 0.262	12.225 ± 0.29
HCT (%)	35.12 ± 0.22	33.82 ± 0.85	32.55 ± 1.35^**∗∗**^	34.2 ± 1.09
PLA ×10^3^/mm^3^	559.25 ± 40.79	543.75 ± 189.50	649.25 ± 113.84	497.75 ± 58.26
%LYM (%)	54.82 ± 23.35	70.87 ± 6.42^*∗*^	63.7 ± 7.44	62.17 ± 3.55
%MON (%)	10.13 ± 5.05	13.72 ± 4.19	13.15 ± 2.92	14.02 ± 1.27
%GRA (%)	24.88 ± 6.70	15.4 ± 3.75^*∗*^	23.15 ± 7.75	23.8 ± 4.75
LYM ×10^3^/mm^3^	8.10 ± 2.20	6.30 ± 0.70^*∗*^	6.05 ± 1.61^*∗*^	5.43 ± 1.03
MON ×10^3^/mm^3^	1.20 ± 0.89	1.22 ± 0.58	1.17 ± 0.22	1.17 ± 0.27
GRA ×10^3^/mm^3^	3.00 ± 1.73	1.55 ± 0.50^*∗*^	2.37 ± 1.26	2.22 ± 0.61

Each value represents the mean ± SEM, *n* = 8. ^*∗*^ *P* < 0.05, ^**∗∗**^ *P* < 0.0.1 compared with control group.
